# The effect of physical exercise on circulating brain‐derived neurotrophic factor in healthy subjects: A meta‐analysis of randomized controlled trials

**DOI:** 10.1002/brb3.2544

**Published:** 2022-03-11

**Authors:** Ya‐Hai Wang, Huan‐Huan Zhou, Qiang Luo, Sidong Cui

**Affiliations:** ^1^ Physical Education College Yunnan Normal University Kunming China; ^2^ School of Public Health Medical College of Soochow University Suzhou China; ^3^ Department of Orthopedics and Traumatology Pu'er Hospital of Traditional Chinese Medicine Pu'er China

**Keywords:** brain‐derived neurotrophic factor, meta‐analysis, neurotrophic factor, physical exercise

## Abstract

**Objective:**

To investigate how physical exercise (PE) would affect brain‐derived neurotrophic factor (BDNF) in randomized controlled trials (RCTs) of healthy subjects.

**Methods:**

Seven databases (PubMed, Web of Science, Cochrane, Embase, PsycINFO, CINAHL, SPORTDiscus) were searched for RCTs assessing the effects of PE on serum and/or plasma BDNF until December 18, 2021. Meta‐analysis was performed by random‐effects method with standardized mean difference (SMD) and 95% confidence intervals (CIs). Subgroup analysis and meta‐regression analysis were conducted to investigate the potential source of heterogeneity. Trim and fill method, and leave‐one‐out cross‐validation were conducted.

**Results:**

Eventually, 21 articles, involving 809 participants, were included in the meta‐analysis. Overall, both acute (5 trials, SMD: 1.20, 95% CI: 0.36 to 2.04, *p* = .005) and long‐term (17 trials, SMD: 0.68, 95% CI: 0.27 to 1.08, *p* = .001) PE had significant positive effects on BDNF levels. Via subgroup analysis, studies of long‐term PE with larger sample sizes, female participants, participants older than 60 years, and aerobic exercise contributed to a more pronounced improvement on BDNF levels than that found when all studies were combined.

**Conclusion:**

Both acute and long‐term PE had significant positive effects on circulating BDNF in healthy subjects. This review suggests that acute exercise and long‐term aerobic exercise are powerful forms of PE to enhance neurotrophic effect, especially for female subjects or subjects over 60 years.

## Graphical abstract

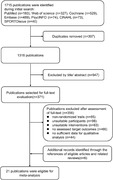

## 1 INTRODUCTION

There is a tremendous amount of evidence that physical exercise (PE) can improve neurological function and counteract the risk of dementia (Larson et al., 2006; Voss et al., 2019). Among the potential mechanisms of PE‐enhancing cognitive effects, neurotrophic molecules such as brain‐derived neurotrophic factor (BDNF) (Erickson et al., 2012) and insulin‐like growth factor‐1, are important candidates. The increased expressions of BDNF (Gomez‐Pinilla et al., 2011; Neeper et al., 1996) were related to the beneficial effect of PE in neurogenesis and neuroplasticity. BDNF, first purified from pig brain (Barde et al., 1982), is a protein of the neurotrophin family promoting proliferation and survival of neurons (Park & Poo, 2013) as well as immunity and tissue repair (Kerschensteiner et al., 1999). BDNF is released by many tissues, including skeletal muscle (J. J. Walsh et al., 2015) in addition to the brain. BDNF plays an essential role in the structure and function of the brain via protecting cells and DNA from damage by down‐regulating oxidative stress (Hacioglu et al., 2016), modulating neurogenesis (Brown et al., 2003), promoting axonal and dendritic growth (Gonçalves et al., 2016), and modulating synaptic plasticity (Zenke et al., 2015). The bulk of available evidences have found that BDNF could improve the cognitive ability of both animal (Vaynman et al., 2004) and human models (Leckie et al., 2014). It was also found that BDNF improved cell signal transduction and restored learning and memory through amyloid‐independent mechanisms in rodent and primate models of Alzheimer's disease (Nagahara et al., 2009).

Existing randomized controlled trials (RCTs) of humans showed that the effects of PE on BDNF are inconsistent, with some finding increases (Rentería et al., 2020; Schmolesky et al., 2013) in BDNF after PE, while most of the others reporting no change (Arrieta et al., 2020; Baird et al., 2018; Forti et al., 2014) in circulating BDNF. This variability may be due to differences in dose parameters, such as type, intensity, and duration of PE. Meta‐analysis (Dinoff et al., 2017) based on pre‐post design showed that an acute bout of PE increases circulating BDNF transiently, while the effect of long‐term PE on neurotrophic molecules is still uncertain. Previous meta‐analyses (Dinoff et al., 2016; Szuhany et al., 2015) on the effect of exercise training on resting concentrations of BDNF in humans found that regular exercise training could enhance the response of BDNF to acute PE. Likewise, their analysis also focused only on estimating the association between exercise and BDNF concentrations through the change of BDNF levels from pre‐exercise to post‐exercise (non‐RCT), which is inefficient in clarifying the actual effect. In addition, one (Szuhany et al., 2015) of these two meta‐analyses included people with diseases that are known to have lower basal BDNF (i.e., Parkinson's disease, obesity, and metabolic syndrome). A recent review by E. I. Walsh et al. (2020) concluded that high‐intensity short‐term activities might effectively promote BDNF response but specific PE type and dose for optimal BDNF release is unclear. Consequently, in the changing context of gradual decline of physical and cognitive abilities in the normal aging process, the magnitude of the actual effects of PE on peripheral BDNF concentrations is still uncertain.

Considering the extensive attention of PE on BDNF and cognition, it is of great significance to collect the existing RCTs for a comprehensive meta‐analysis to determine: (1) the specific role of PE on BDNF under physiological condition for healthy subjects and (2) how do training protocols and characteristics of subjects influence the outcomes.

## 2 METHODS

### 2.1 Literature search

The systematic search was carried out following the guidelines of Preferred Reporting Items for Systematic Reviews and Meta‐Analysis (PRISMA) in this meta‐analysis (Moher et al., 2009). Seven electronic databases, including PubMed, Web of Science, Cochrane Library, Embase, PsycINFO, Cumulated Index to Nursing and Allied Health Literature (CINAHL), and SPORTDiscus, were searched from 1980 to December 8, 2021 for relevant articles, using the following search strategy: (Exercise OR “Physical Exercise” OR “Exercise Therapy”) AND (“Brain‐Derived Neurotrophic Factor” OR BDNF) AND “randomized controlled trial.” Detailed search strategy was shown in Table S1.

### 2.2 Study selection

Two researchers (YHW and HHZ) independently screened titles and abstracts, then reviewed full‐text for eligibility. The third researcher (SDC) arbitrated any discrepancies to reach consensus. We also conducted a manual search for references to eligible articles, relevant review articles, and systematic reviews. For selection, studies had to fulfill the following criteria: (1) being human RCTs with parallel or crossover; (2) the volunteers were healthy people; (3) using PE as the intervention treatment; the comparisons were exercise versus nonexercise control or exercise plus other intervention versus other intervention only; (4) the interested outcomes were BDNF in plasma or serum; and (5) have been published in English since 1980.

The exclusion criteria were (1) studies including people with diseases; (2) lacking net changes of neurotrophic biomarkers and their corresponding SDs as outcome measures or providing sufficient information to calculate them (mean changes of treatment [both intervention groups and control groups] ± SD).

### 2.3 Data extraction and quality assessment

The study selection, data extraction, and quality assessment were undertaken independently by two investigators (YHW and HHZ) with standard form. Eligible studies were reviewed and the following data were extracted: The first author's surname, publication year, study design, study location, sample size, participants age and gender, baseline body mass index (BMI) of participants, PE intervention (duration, type, intensity, frequency), and reported circulating BDNF levels. If one study contained two or more independent intervention strata (e.g., different types, intensity, frequency or duration of PE), it was treated as separate trials for analysis. All types of PE (i.e., aerobic exercise, resistance exercise, and multicomponent exercise) were included in this review. Intensity of exercise was classified by maximal heart rate (HRmax), maximal oxygen uptake (VO_2_peak), and repetition maximum (RM) according to American College of Sports Medicine (Haskell et al., 2007). Advanced data extraction was performed using Adobe Photoshop for studies that did not directly provide data but present their data in a graphic format, according to the protocol proposed by Gheibi et al. (2019). The methodological quality of selected studies was evaluated by the PEDro scale (Maher et al., 2003). The Grading of Recommendation, Assessment, Development and Evaluation (GRADE) system was used to assess the evidence level of each outcome (Goldet & Howick, 2013). According to the specific regulations of GRADE guidelines, study design dictates baseline quality of the evidence (RCTs are initially defined as high quality) but other factors could decrease (e.g., unexplained heterogeneity) or increase (e.g., a large magnitude of effect) the quality level (Goldet & Howick, 2013). Discrepancies were resolved by discussing with the third reviewer (SDC) to reach consensus.

### 2.4 Statistical analysis

The pooled effect sizes were defined as the standardized mean difference (SMD) with 95% confidence intervals (CIs) of net changes of the concentrations of BDNF. The heterogeneity among studies was evaluated using *I* ^2^ and Cochrane's *Q* test and there is heterogeneity when *I* ^2^ > 50% and *p‐*value < .1 for *Q* test (Higgins et al., 2003). Considering the existing heterogeneity between studies, the random effects model was used in pooling estimates of net changes. For studies in which either baseline or final mean and standard deviation (SD) of outcomes were not provided directly, advanced data extraction using the reported method proposed by Wan et al. (2014) was conducted.

Firstly, a primary meta‐analysis was conducted to establish the overall effect. Then subgroup analyses were performed to investigate the potential source of heterogeneity based on the sample size, region, gender, age, baseline BMI of participants, duration of intervention, and type and intensity of exercise (only conducted if more than five trials reported the same outcomes). Differences between groups and sources of heterogeneity were tested by meta‐regression analysis, with *p*‐value < .1 as statistically significant.

Both Begg's and Egger's regression tests as well as funnel plots were utilized to assess the publication bias, with a *p*‐value < .1 suggesting the presence of bias (Egger et al., 1997). If publication bias was encountered, the trim and fill method was performed (Duval & Tweedie, 2000). Sensitivity analysis using leave‐one‐out method was performed to investigate key studies that have substantial impact on the heterogeneity between studies (Serban et al., 2015), using *p* < .1 as the criterion. All analyses were performed using STATA version 11.0 (Stata Corp, College Station, TX, USA), with double data input to avoid input errors. *p* < .05 was deemed as statistically significant unless specified elsewhere.

## 3 RESULTS

### 3.1 Flow of study selection

**FIGURE 1:**
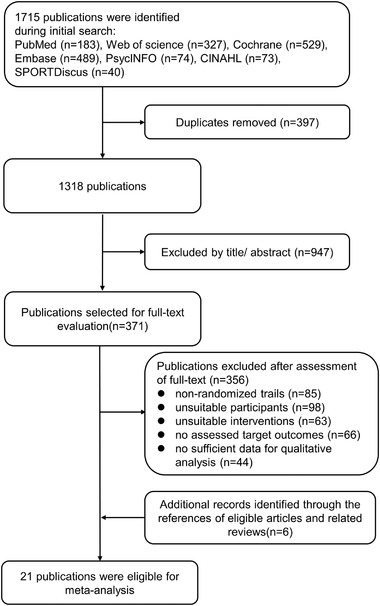
Flowchart of study selection through the review

The detailed flowchart of literature search and study selection is presented in Figure 1. A total of 1658 articles (183 from PubMed, 327 from web of science, 529 from Cochrane library, 489 from Embase, 74 from PsycINFO, 73 from CINAHL, and 40 from SPORTDiscus) were initially identified from the databases search. After excluding the duplicates and screening the titles and abstracts, 371 articles were left for full‐text review, of which 356 articles were further eliminated for the following reasons: 85 articles were non‐RCT design, participants of 98 studies were not healthy, 63 articles had improper intervention or control, assessable target outcomes were not reported in 66 articles, and 44 articles lack sufficient data for quantitative analysis. Additionally, reference lists of all eligible articles and relevant reviews (Azevedo et al., 2020; Dinoff et al., 2017; Marinus et al., 2019; Stigger et al., 2019) were screened and identified six eligible articles. Finally, 21 eligible studies involving 809 participants met inclusion criteria for final meta‐analysis.

### 3.2 Qualities of included studies and outcome measure evidences by GRADE

The methodological qualities of one study (Matura et al., 2017) was rated as excellent quality according to the PEDro scale (scores ≥ 9), while the remaining 20 studies were good quality (6‐8 scores) mostly because of lacking blindness (Table 1). The evidence quality of acute PE on BDNF levels was at a moderate level, while the quality of long‐term PE on BDNF levels was at a low level according to the GRADE system (Table S2).

**TABLE 1:** Characteristics of included studies in this meta‐analysis (21 studies)

Study	Design	Country	Subject	Comparators	Interventions	Duration of intervention	BDNF measure	Quality^a^
**BDNF measured after acute exercise**								
Arazi et al., 2021	RP	Iran	Healthy older men (***N* = 30**)	**Control (*N* = 10)**: Did not perform any exercise.	**Resistance Exercise (*N* = 10)**: warm‐up (10 min); 6 exercises with 10 repetitions (30 min); cool‐down (5 min). *Intensity: *65−70% of 1RM **Aerobic Exercise (*N* = 10)**: warm‐up (10 min); running (3 × 10‐min with 120 s interval); cool‐down (5 min). *Intensity*: 65−75% of HRmax	45 min	Serum	Good
Helm et al., 2017	RP	USA	Neurologicaly intact subjects (***N* = 54**)	**Rest (*N* = 27)**: quiet rest.	**Resistance Exercise (*N* = 27)**: pedaling with high resistance (1 min); pedaling with resistance decreased by half (1 min); rest (1 min); pedaling with high resistance (1 min); pedaling with resistance decreased by half (1 min) *Intensity: *> 80% HRmax	5 min	Serum	Good
Schmolesky et al., 2013	RP	USA	Healthy adult males (***N* = 45**)	**Control (*N* = 10)**: Remained seated and at rest during the exercise period.	**Aerobic Exercise (*N* = 35)**: cycling (30 min). *Intensity*: 60–80% of HR reserve.	30 min	Serum	Good
Urzi et al., 2019 ^b^	RP	Slovenia	Healthy elderly women (***N* = 20**)	**Control (*N* = 9)**: No changes in diet habits	**Resistance Exercise (*N* = 11)**: warm‐up (10 min); 8 resistance exercises (35–40 min). *Intensity*: moderate	45‐50 min	Plasma	Good
Winter et al., 2007	RC	Germany	Male healthy sport students (***N* = 27**)	**Relaxed (*N* = 27)**: Being sedentary.	**Aerobic Exercise (*N* = 27)**: low impact running (40 min) *Intensity*: blood lactate level ≤ 2 mmol); **Resistance Exercise (*N* = 27)**: 2 sprints of 3 min at increasing speed *Intensity*: blood lactate level > 10 mmol *Wash‐out*: at least 1 week apart	40 min	Serum	Good
**BDNF measured after long‐term exercise**								
Arrieta et al., 2020	RP, Sb	Spain	Men and women living in nursing homes (***N* = 88**)	**Control (*N* = 45)**: Engaged in routine activities.	**Multicomponent Exercise (*N* = 43)**: warm‐up (5 min); strength training, balance exercises, proprioceptive exercises, and stepping practice; deep breathing exercise (5 min). walking (5‐20 min/day). *Intensity*: 40−70% of 1RM *Frequency*: 2 sessions/week (except for walking)	24 week	Serum	Good
Čekanauskaitė et al., 2020	RP	Lithuania	Physically inactive healthy older men (***N* = 33**)	**Control (*N* = 15)**: Maintain their daily living habits.	**Aerobic Exercise (*N* = 18)**: warm‐up (15 min); yoga asanas (45 min), Himalayan kriya breathing exercises (25 min); relaxation in shavasana (corpse pose) (15 min). *Intensity*: n/a *Frequency*: 2 sessions/week	10 week	Serum	Good
Cho & Roh, 2019	RP	South Korea	Healthy women aged 65 years or older (***N* = 37**)	**Control (*N* = 18)**: Maintained their activities of daily living.	**Aerobic Exercise (*N* = 19)**: warm‐up and cool‐down (10 min); taekwondo training (50 min) *Intensity*: 50−80% of HRmax *Frequency*: 5 sessions/week	16 week	Serum	Good
Forti et al., 2014	RP	Belgium	Elderly volunteers (***N* = 40**)	**Control (*N* = 20)**: Maintain daily activity levels.	**Resistance Exercise (*N* = 20)**: warm‐up; progressive strength training; muscle stretching (60 min). *Intensity*: 50–80% of 1RM *Frequency*: 3 sessions/week	12 week	Serum	Good
Goekint et al., 2010	RP	Belgium	Untrained subjects (***N* = 23**)	**Control (*N* = 8)**: Remained physically inactive.	**Resistance Exercise (*N* = 15)**: warm‐up; six strength exercises (chest press, shoulder press, vertical traction, leg press, adductor strength, and abductor strength). *Intensity*: 50−80% 1RM *Frequency*: 3 sessions/week	10 week	Serum	Good
Jeon & Ha, 2015	RP	South Korea	Healthy junior high school students (***N* = 20**)	**Control (*N* = 10)**: Continue their daily normal and sedentary activities.	**Aerobic Exercise (*N* = 10)**: treadmill exercise until burned 200 kcal. *Intensity*: 40−60% of VO_2_max *Frequency*: 3 days/week	8 week	Serum	Good
Jeon & Ha, 2017	RP	South Korea	Male middle school students **(*N* = 40)**	Stretching group **(*N* = 10)**: Performed whole‐body stretching at the same time.	**Aerobic Exercise (*N* = 30)**: treadmill exercise until burned 200 kcal. Intensity: 40% (low intensity group, *N* = 10) / 55% (moderate intensity group, *N* = 10) / 70% (high intensity group, *N* = 10) of VO2max. Frequency: 4 times/week	12 week	Serum	Good
Kim & Kim, 2018	RP	South Korea	Sedentary elderly women **(*N* = 26)**	**Control (*N* = 12)**: Make no changes to their diet and exercise habits.	**Aerobic Exercise (*N* = 14)**: warm‐up (10 min); training (40 min); cool‐down (10 min). Intensity: 40−50% (1−4 week), 50−60% (5−8 week), 60−65% (9−12 week), and 65−70% (13−16 week) of HR reserve Frequency: 2 times/week	16 week	Serum	Good
Ledreux et al., 2019	RP	USA & Sweden	Healthy older individuals **(*N* = 68)**	**Control (*N* = 39)**: Not described.	**Aerobic Exercise (*N* = 29)**: aerobic exercise training following pre‐recorded video segments (35 min) Intensity: n/a Frequency: 5 days/week	5 week	Serum	Good
Maass et al., 2016	RP	Germany	Sedentary healthy older adults **(*N* = 40)**	**Control (*N* = 19)**: Supervised progressive muscle relaxation/stretching training (45 min). Frequency: 2 times/week	**Aerobic Exercise (*N* = 21)**: warm‐up (5 min); training (40 min); stretching (5 min). Intensity: 65–80% of HRmax Frequency: 3 days/week	12 week	Serum, plasma	Good
Matura et al., 2017	RP, Sb	Germany	Healthy older participants **(*N* = 53)**	**Control (*N* = 24)**: Not to change their habitual physical activity.	**Aerobic Exercise (*N* = 29)**: supervised cycle ergometer training (30 min). Intensity: 64 ± 9% of VO2max Frequency: 3 sessions/week	12 week	Serum	Excellent
Nilsson et al., 2020	RP	Sweden	Healthy older adults** (*N* = 70)**	**Control (*N* = 21)**: Seated rest.	**Aerobic Exercise (*N* = 49)**: warm‐up (5 min); aerobic activity (30 min). Intensity: 65−75% of HRmax Frequency: 3 days/week	12 week	Serum	Good
Rentería, 2020	RP	Mexico	Healthy young adult women **(N = 17)**	**Control (*N* = 8)**: Maintain their regular physical activity habits.	**Aerobic Exercise (*N* = 9)**: warm‐up; 3–5 cycling bouts of 30s+4‐min recovery. Intensity: 80% maximal aerobic power Frequency: 3 days/week	4 week	Serum	Good
Schiffer, 2009	RP	Germany	Healthy sports students **(*N* = 36)**	**Control (*N* = 18)**: Continue their regular lifestyle.	**Resistance Exercise (*N* = 9)**: 3 sets of 8–10 repetition of complete body work out. Intensity: 80% of 1RM Frequency: 3 times/week **Aerobic Exercise (*N* = 9)**: ran (45 min). Intensity: 80% of HRmax Frequency: 3 times/week	12 week	Plasma	Good
Seifert et al., 2010	RP	Denmark	Sedentary male **(*N* = 12)**	**Control (*N* = 5)**: Continue sedentary lifestyle; on a diet creating a negative energy balance of ∼600 kcal/day.	**Aerobic Exercise (*N* = 7)**: cycling or running or swimming (60 min or until 600 kcal expenditure was reached). Intensity: 70% of HRmax Frequency: everyday	12 week	Plasma	Good
Solianik et al., 2021	RP	Lithuania	Healthy elderly **(*N* = 30)**	**Control (*N* = 15)**: Maintain their daily routines.	**Aerobic Exercise (*N* = 15)**: warm‐up (15 min); 8‐form Yang‐style tai chi (40 min); cool‐down (5 min). Intensity: n/a Frequency: 2 times/week	10 week	Serum	Good
Urzi et al., 2019 ^b^	RP	Slovenia	Healthy elderly women **(*N* = 20)**	**Control (*N* = 9)**: No changes in diet habits.	**Resistance Exercise (*N* = 11)**: warm‐up (10 min); 8 resistance exercises (35–40 min). Intensity: moderate	12 week	Plasma	Good

*Abbreviations*: BDNF, brain‐derived neurotrophic factor; HRmax, maximal heart rate; kcal, kilocalories; km, kilometer; min, minute; RC, randomized crossover; RM, repetition maximum; RP, randomized‐parallel; s, second; Sb, single blind; SB, single‐blinded; VO2max, maximal oxygen uptake; wk, week.

^a^ Better methodological quality is indicated by a higher PEDro score (9–10: excellent; 6–8: good; 4–5: fair; < 4: poor).

^b^ The study examined both acute and chronic effects of PE on BDNF levels.

### 3.3 Characteristics of included studies

Table 1 summarizes the characteristics of the included 21 studies. The final sample consisted of 809 unique participants, with mean age ranging from 15 to 84.9. Sample sizes ranged from 12 to 88 participants, with a median size of 36.5. The average baseline BMI value of participants ranged from 17.2 to 28.5. Fifteen studies reported gender composition of the participants, and 57.2% of the participants were male. These studies were carried out in different countries including Germany (*n* = 4), South Korea (*n* = 4), USA (*n* = 3), Belgium (*n* = 2), Lithuania (*n* = 2), Sweden (*n* = 2), and other five countries with single study. Different types (aerobic exercise: 16 trials; resistance exercise: 8 trials; multicomponent exercise: 1 trials) of PE were reported in those included studies. In addition, the intervention duration of long‐term PE ranged from 4 to 24 weeks.

### 3.4 Effect of acute PE on BDNF levels

**FIGURE 2:**
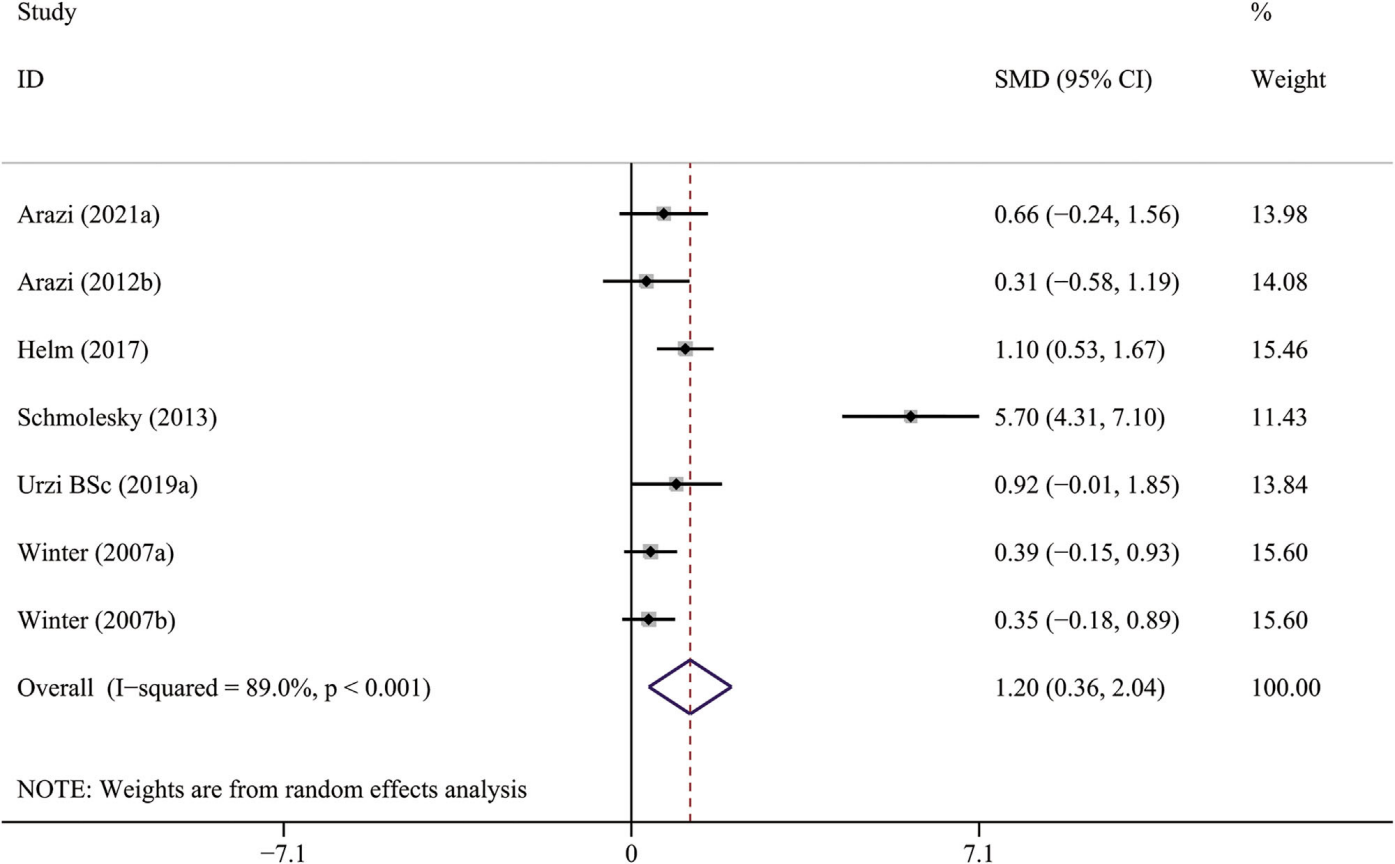
The forest plot of acute PE intervention on circulating BDNF levels

Five studies (Arazi et al., 2021; Helm et al., 2017; Schmolesky et al., 2013; Urzi et al., 2019; Winter et al., 2007) examined the effect of acute PE on BDNF levels. The results of analysis showed that acute PE remarkably elevated the levels of BDNF (SMD: 1.20, 95% CI: 0.36 to 2.04, *p* = .005), with a high heterogeneity observed (*p* < .001, *I* ^2 ^= 89.0%) (Table 2; Figure 2).

**TABLE 2:** Results of subgroup analysis and publication bias stratified by study characteristics

				Heterogeneity			*p* ^4^	
Outcomes	Trials	SMD (95% CI)	*p* ^1^	*I* ^2^ (%)	*p* ^2^	*p* ^3^	Begg's value	Egger's value
**BDNF, ng/ml (acute effect)**	5	1.20 (0.36, 2.04)	**.005**	**89.0**	**<.001**		–	–
**BDNF, ng/ml (long‐term effect)**	17	0.68 (0.27, 1.08)	**.001**	82.8	**<.001**		**.030**	.473
**Sample size**						.890		
**≤ 20**	6	0.33 (−0.12, 0.77)	.150	48.9	**.048**		.210	.321
**> 20**	11	0.93 (0.34, 1.52)	**.002**	89.1	**<.001**		**.008**	**.073**
**Region**						.581		
Conducted in Asia	4	0.56 (0.05, 1.07)	**.003**	54.6	**.051**		–	–
Conducted in Europe	11	0.74 (0.11, 1.37)	**.021**	88.6	**<.001**		.193	.561
Conducted in North American	1	1.26 (0.21, 2.31)	**.019**	–	**–**		–	–
**Gender**						**.081**	–	–
Male	3	0.35 (−0.07, 0.76)	.104	0	.485		–	–
Female	4	1.10 (0.53, 1.68)	**<.001**	42.0	.160		–	–
**Age**						.287		
≤ 60 years	6	0.30 (−0.12, 0.73)	.163	45.4	**.066**		.348	.313
**>** 60 years	11	0.95 (0.35, 1.55)	**.002**	89.2	**<.001**		**.020**	**.071**
**Baseline BMI**						.340		
< 25	4	0.42 (−0.03, 0.88)	.070	20.2	.286		–	–
≥ 25	8	0.65 (0.07, 1.24)	**.029**	85.8	**<.001**		**.035**	.509
**Duration**						.447		
≤ 8 weeks	3	0.62 (0.05, 1.20)	**.034**	39.1	.193		–	**–**
8 < duration ≤ 12 weeks	11	0.66 (0.09, 1.24)	**.025**	86.1	**<.001**		.108	.826
> 12 weeks	3	−0.71 (−0.24, 1.66)	.141	84.7	**.001**		–	–
**Type of exercise**						.169		
Aerobic exercise	13	0.86 (0.37, 1.36)	**.001**	83.5	**<.001**		**.030**	.473
Resistance exercise	4	0.20 (−0.59, 0.98)	.626	71.3	**.015**		–	–
Multicomponent exercise	1	–0.01 (−0.43, 0.41)	.960	–	–		–	–
**Intensity of exercise**						.915		
Low	3	0.14 (−0.29, 0.58)	.518	21.8	.278		–	–
Moderate	4	0.75 (−0.79, 2.29)	.339	91.5	**<.001**		–	–
High	3	0.30 (−0.63, 1.23)	.523	65.0	.058		**–**	**–**

*Note: p* ^1^ value for net change; *p* ^2^ value for heterogeneity in the subgroup; *p* ^3^ value for heterogeneity between groups with meta‐regression, analyzed as categorical variables; *p* ^4^ value for publication bias (conducted only when trials > 5); significant *p*‐values are highlighted in bold prints.

*Abbreviations*: BDNF, brain‐derived neurotrophic factor; BMI, body mass index.

### 3.5 Effect of long‐term PE on BDNF levels

**FIGURE 3:**
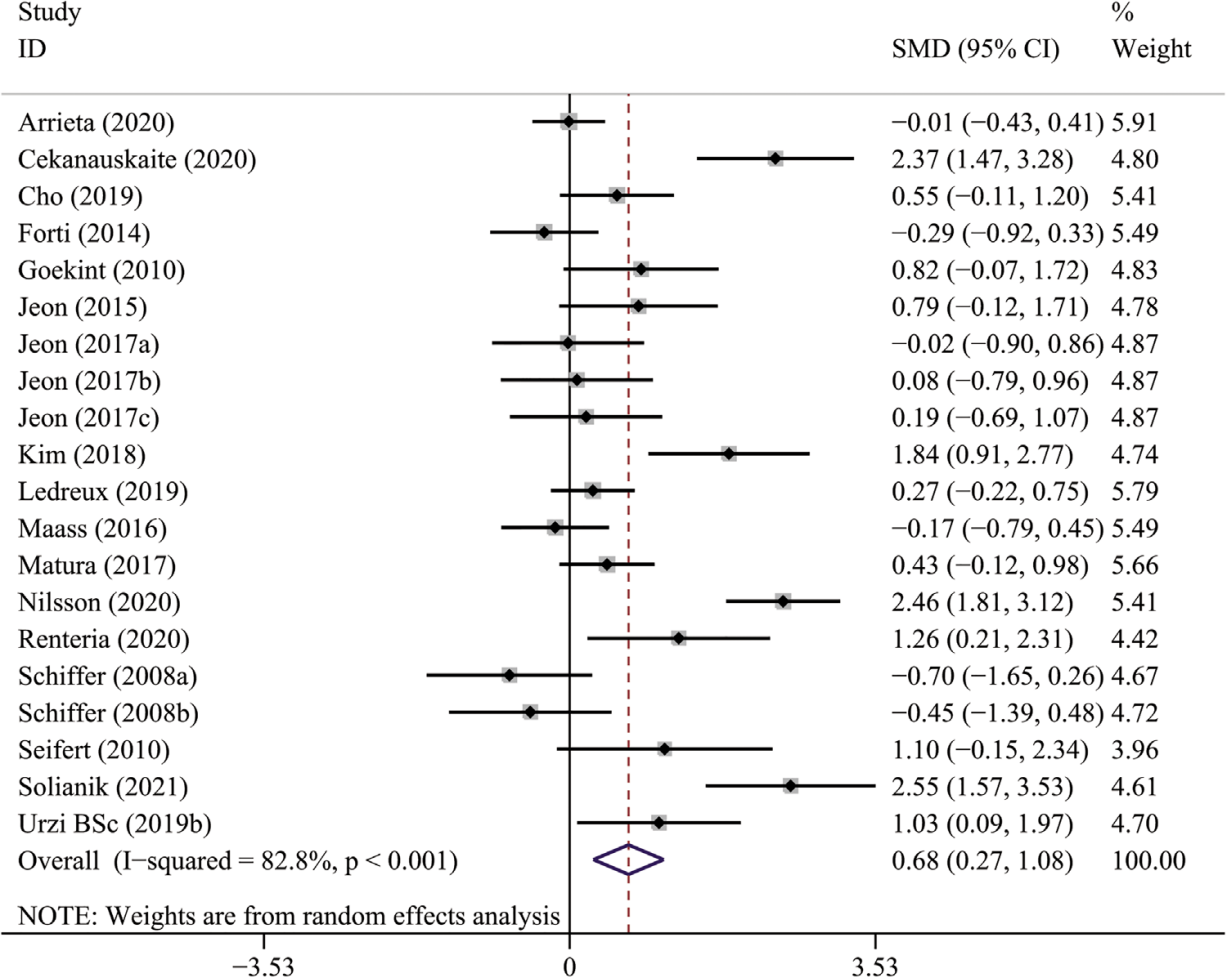
The forest plot of long‐term PE intervention on circulating BDNF levels

Seventeen studies (Arrieta et al., 2020; Čekanauskaitė et al., 2020; Cho & Roh, 2019; Forti et al., 2014; Goekint et al., 2010; Jeon & Ha, 2015, 2017; Kim & Kim, 2018; Ledreux et al., 2019; Maass et al., 2016; Matura et al., 2017; Nilsson et al., 2020; Rentería et al., 2020; Schiffer et al., 2009; Seifert et al., 2010; Solianik et al., 2021; Urzi et al., 2019) that reported the effect of long‐term PE on BDNF were included in the meta‐analysis. The primary meta‐analysis revealed that long‐term PE could significantly increase BDNF levels (SMD: 0.68, 95% CI: 0.27 to 1.08, *p* = .001), with a high heterogeneity observed (*p* = .000, *I* ^2 ^= 82.8%) (Table 2; Figure 3).

#### 3.5.1 The results of subgroup analysis

Subgroup analysis revealed that the pooled effect of long‐term PE on BDNF concentration was related to sample size, gender, age, baseline BMI, duration of PE, and type of PE. A significantly positive effect on BDNF levels was observed only in studies with larger sample sizes (*n* > 20) (11 trials, SMD: 0.93, 95% CI: 0.34 to 1.52, *p* = .002) but not in studies with smaller sample sizes (*n* ≤ 20) (6 trials, SMD: 0.33, 95% CI: −0.12 to 0.77, *p* = .150) (Figure 4A). It also indicated that the effect of PE intervention on BDNF levels was significant only in female participants (4 trials, SMD: 1.10, 95% CI: 0.53 to 1.68, *p* < .001) (Figure 4B). In addition, PE intervention remarkably elevated BDNF levels in participants over 60 years (11 trials, SMD: 0.95, 95% CI: 0.35 to 1.55, *p* = .002), whereas not in participants younger than 60 years (Figure 4C). Subgroup analysis based on baseline BMI of participants showed that BDNF levels significantly increased only in participants whose baseline BMI ≥ 25 (8 trials, SMD: 0.65, 95% CI: 0.07 to 1.24, *p* = .029), but not in participants whose baseline BMI < 25 (4 trials, SMD: 0.42, 95% CI: −0.03 to 0.88, *p* = .070) (Figure 4D). Significant improvements on BDNF concentrations were observed only in studies lasting less than 8 weeks (3 trials, SMD: 0.56, 95% CI: 0.01 to 1.12, *p* = .041) or between 8 and 12 weeks (11 trials, SMD: 0.47, 95% CI: 0.19 to 0.76, *p* = .001), but not in studies lasting more than 12 weeks (3 trials, SMD:1.34, 95% CI: −0.07 to 2.76, *p* = .063) (Figure 5A). Only aerobic exercise significantly elevated the levels of BDNF (13 trials, SMD: 0.86, 95% CI: 0.37 to 1.36, *p* = .001), while resistance exercise (4 trials, SMD: 0.20 95% CI: −0.59 to 0.98, *p* = .626) or multicomponent exercise (1 trials, SMD: −0.01, 95% CI: −0.43 to 0.41, *p* = .960) did not (Figure 5B).

FIGURE 4The subgroup analysis of long‐term PE intervention on circulating BDNF levels stratified by sample size (A), gender (B), age (C), and baseline BMI (D)

**Figure:**
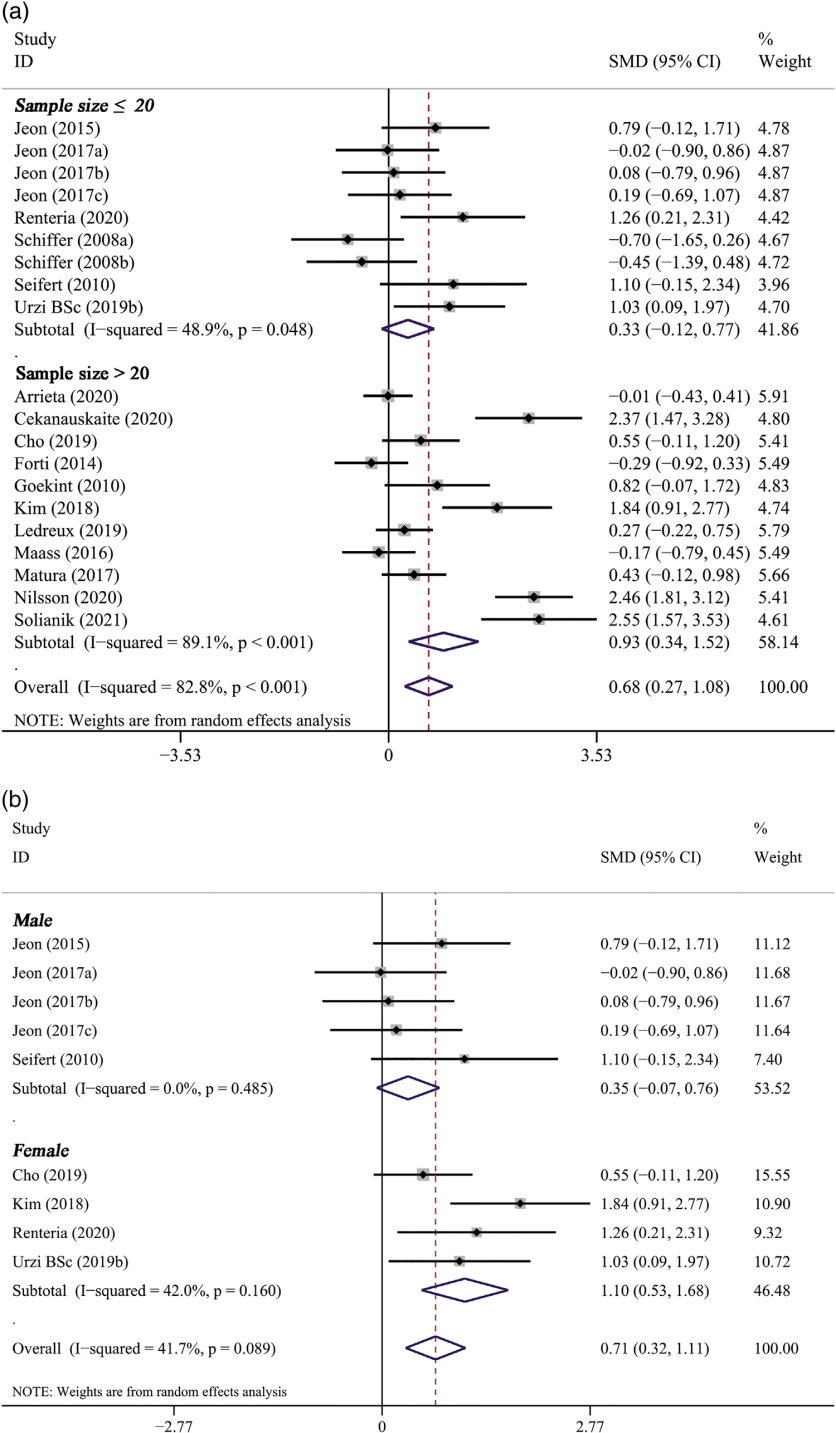

**Figure:**
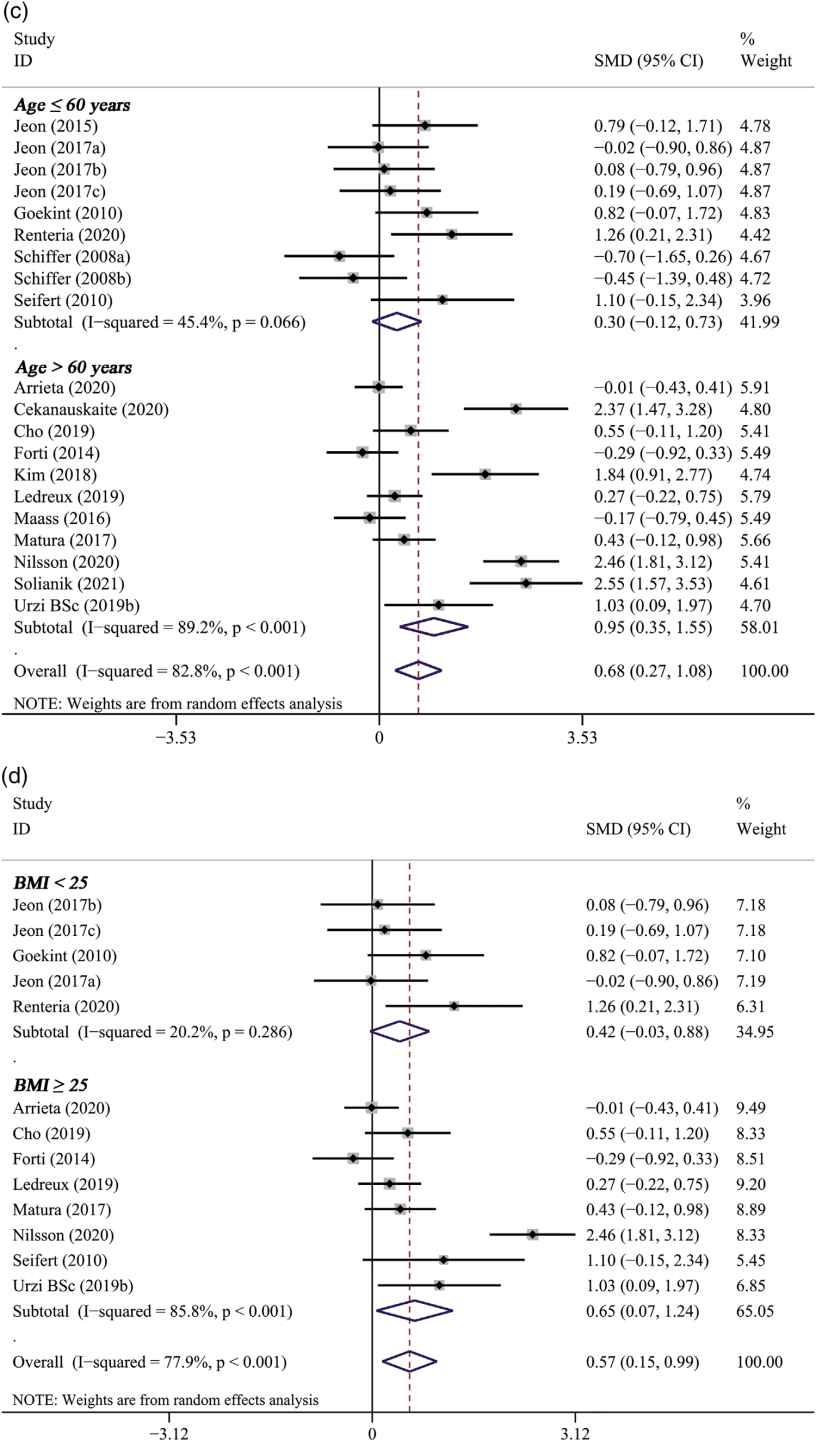

FIGURE 5The subgroup analysis of long‐term PE intervention on circulating BDNF levels stratified by duration of exercise (A) and type of exercise (B)

**Figure:**
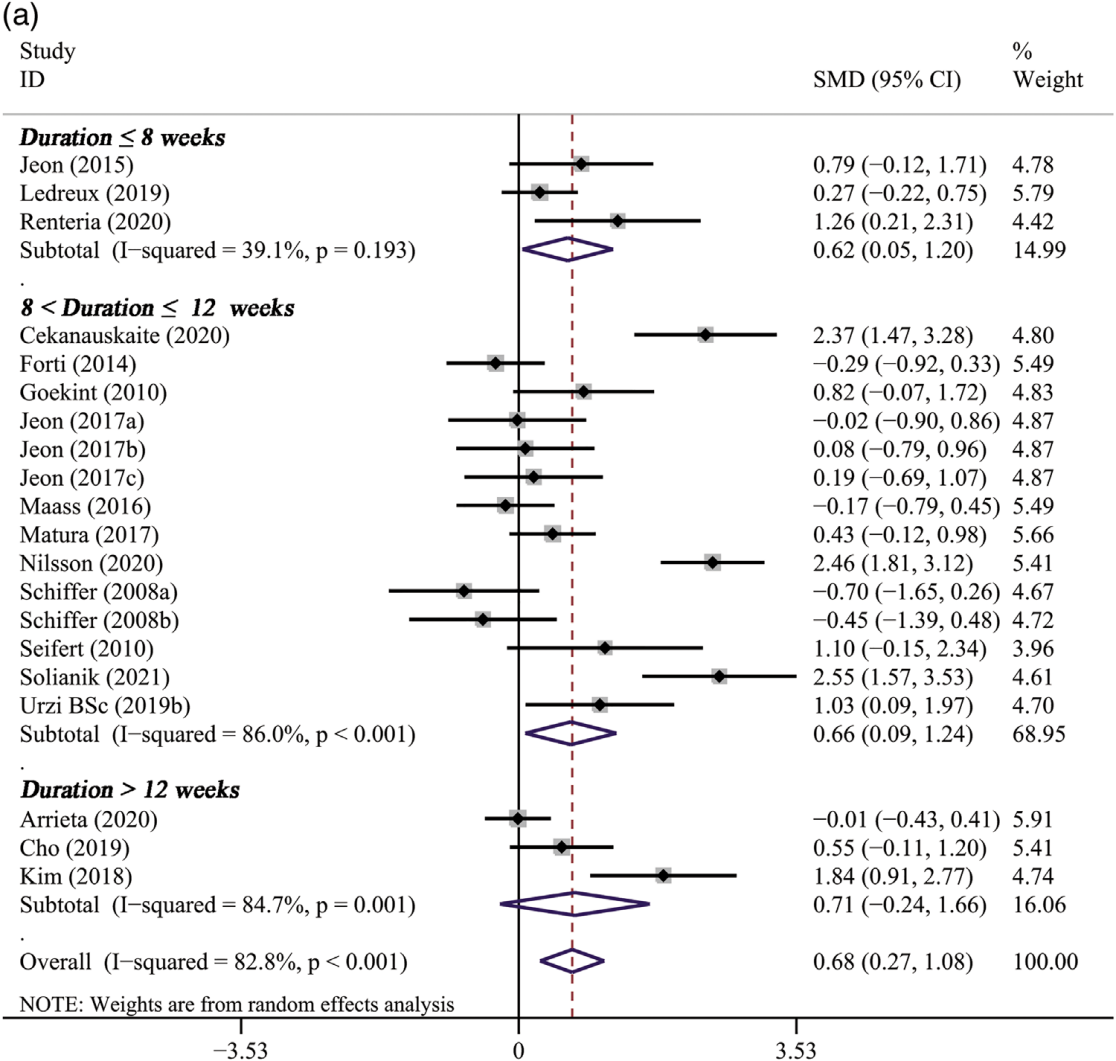

**Figure:**
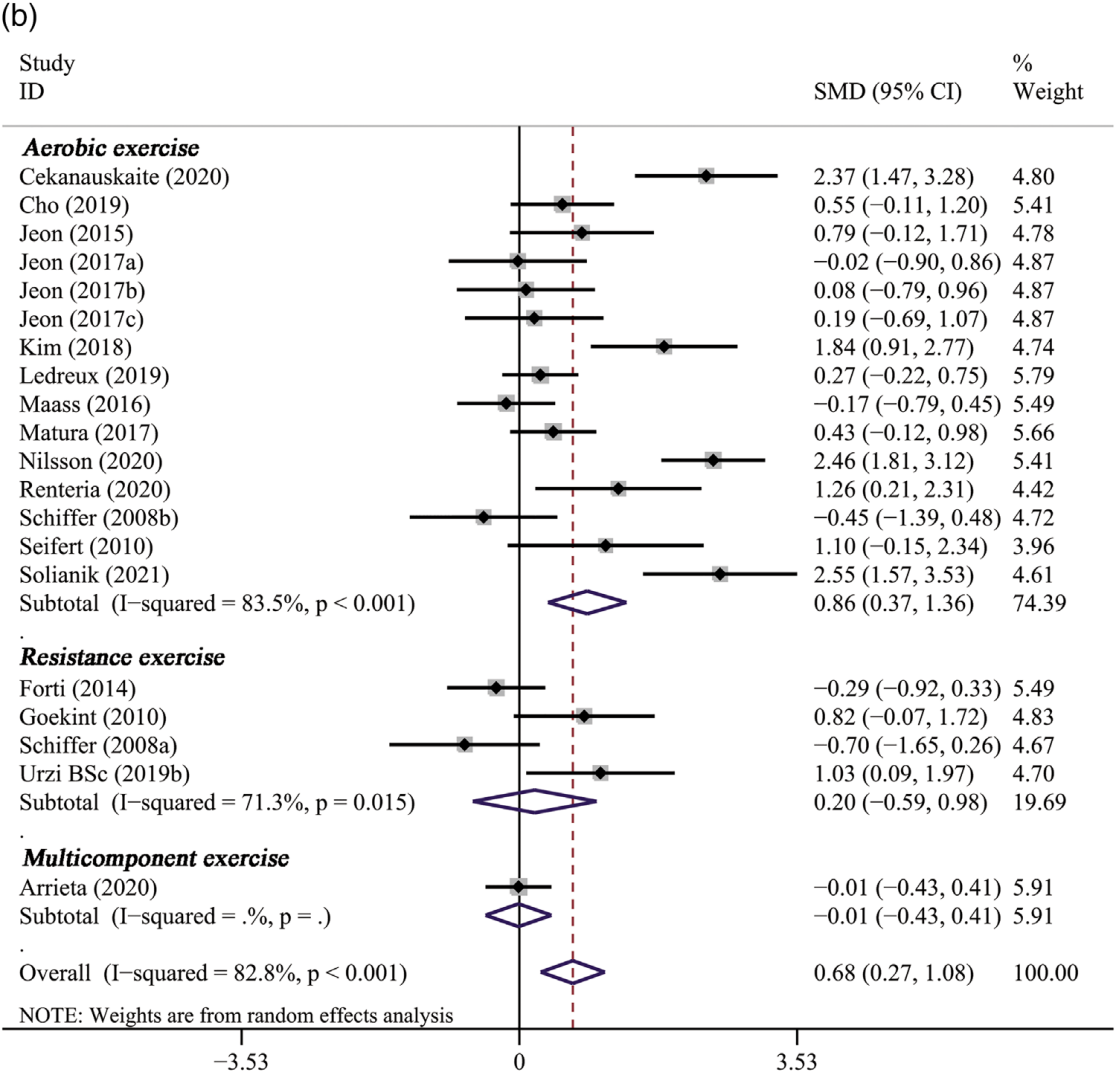

#### 3.5.2 The results of meta‐regression

Meta‐regression analysis was conducted to explore the potential sources of heterogeneity. Among selected covariates, including sample size, region, gender, age, baseline BMI, duration of PE, type of PE, and intensity of PE, the results of meta‐regression analysis revealed that gender of participants was a potential confounder of the effect of long‐term PE intervention on the BDNF levels, with adjusted *R* ^2^ of 59.01% (*p* = .081) (Table 2).

#### 3.5.3 Publication bias

Publication bias was suggested by Begg's test (*p* = .030), but not by Egger's test (*p* = .473) in the primary meta‐analysis (Table 2). Evidences also showed publication bias in the subgroup results of sample size > 20, age ≥ 60 years, baseline BMI ≥ 25, and aerobic exercise (Table 2). As shown in Table 3, for those results with publication bias indicated by Begg's and Egger's tests, the pooling estimates were recalculated using Duval and Tweedie's trim and fill method. The results of meta‐analyses remained unchanged or still statistically significant after being adjusted by trim and fill method, which confirmed and strengthened the evidence‐base regarding the effects of long‐term PE on BDNF (Table 3).

**TABLE 3:** Trim and fill analysis and leave‐one‐out analysis

		SMD (95% CI)		*p*		
Trim and fill analysis	Trials (*n*)	Before adjusted	After adjusted	Before adjusted	After adjusted	Adjusted studies
**BDNF, ng/ml (Long‐term effect)**	17	0.68 (0.27, 1.08)	Unchanged	**.001**	Unchanged	–
Sample size > 20	11	0.93 (0.34, 1.52)	Unchanged	**.002**	Unchanged	–
Age ≥ 60 years	11	0.95 (0.35, 1.55)	0.80 (0.17, 1.44)	**.002**	**.014**	3
BMI ≥ 25	8	0.65 (0.07, 1.24)	Unchanged	**.029**	Unchanged	–
Aerobic exercise	15	0.86 (0.37, 1.36)	Unchanged	**.001**	Unchanged	–

		SMD (95% CI)		*P*		
Leave‐one‐out cross validation	Trials (*n*)	Before adjusted	After adjusted	Before adjusted	After adjusted	Omitted studies
**BDNF, ng/ml (Acute effect)**	5	1.20 (0.36, 2.04)	0.60 (0.32, 0.88)	**.005**	**<.001**	1
**BDNF, ng/ml (Long‐term effect)**	17	0.68 (0.27, 1.08)	0.38 (0.13, 0.62)	**.001**	**.003**	7

*Note*: Significant *p*‐values are highlighted in bold prints.

*Abbreviations*: BDNF, brain‐derived neurotrophic factor; BMI, body mass index; CI, confidence interval.

### 3.6 Sensitivity analysis

Regarding the robustness of overall effect sizes, we performed leave‐one‐out cross validation for sensitivity analysis. The results of leave‐one‐out cross validation suggested that one study in acute PE (Schmolesky et al., 2013) and seven studies in long‐term PE on BDNF (Arrieta et al., 2020; Čekanauskaitė et al., 2020; Forti et al., 2014; Kim & Kim, 2018; Nilsson et al., 2020; Schiffer et al., 2009; Solianik et al., 2021) contributed to the 96.2% and 73.2% of heterogeneity between studies, respectively. After excluding the mentioned trials, the pooled results of acute PE (SMD: 0.60, 95% CI: 0.32 to 0.88, *p* < .001, *I* ^2 ^= 3.8%) and long‐term PE (SMD: 0.38, 95% CI: 0.13 to 0.62, *p* = .006, *I* ^2 ^= 22.2%) remained significant elevated (Table 3).

## 4 DISCUSSION

This is the first meta‐analysis based on RCTs but not pre‐post design to estimate the effect of PE (both acute and long‐term) on the levels of circulating BDNF among healthy subjects. Overall, our present results mainly revealed that both acute and long‐term PE significantly increased BDNF levels, although study heterogeneity was very high for both acute and long‐term effects. Furthermore, studies of long‐term PE with larger sample sizes, female participants, participants older than 60 years, and aerobic exercise contributed to a more pronounced improvement on BDNF levels than that found when all studies were combined.

Acute PE is an effective stimulating factor to increase peripheral BDNF. A bulk of available evidence has reported that acute PE is associated with increased circulating BDNF (Dinoff et al., 2017; Szuhany et al., 2015). H. C. Cho et al. (2012) reported that progressive, maximum intensity treadmill exercise increased the entire peripheral BDNF levels including serum, plasma, and platelets. The release of BDNF from platelets can be changed by allergic airway inflammation (Lommatzsch, Schloetcke, et al., 2005), so acute PE may affect the release of BDNF from platelets by stimulating inflammatory response (Scheffer & Latini, 2020). Moreover, the transient BDNF response to acute exercise may induce a series of neuronal responses to improve cognitive function (Bechara, Lyne, & Kelly, 2014).

Overall, and in accordance with other studies (Dinoff et al., 2016; Szuhany et al., 2015), we observed a significant elevated effect of long‐term PE on BDNF levels. One of the possible mechanisms by which long‐term PE induced brain plasticity and cognitive enhancement is via stimulating an increase in the concentration of BDNF (Gligoroska & Manchevska, 2012). Usually, 99% of BDNF in circulation binds to platelets (E. I. Walsh et al., 2020), which are stored in the spleen for later release. After BDNF is released into plasma, it can bind with specific neural receptors (E. I. Walsh et al., 2020). PE can stimulate the release of BDNF from brain, skeletal muscle, platelets, and other tissues (J. J. Walsh & Tschakovsky, 2018), mainly by increasing blood circulation throughout the body and the release of platelets from the spleen (E. I. Walsh et al., 2020). Due to differences in the sample size of the included studies (ranged from 12 to 88), we stratified the results according to the sample size. Our subgroup analysis revealed that only studies with larger sample sizes displayed significant elevation in BDNF levels post long‐term PE. Small sample size studies may have sampling error and instability, and are more likely to draw false negative conclusions, namely type Ⅱ error (Akobeng, 2016). However, after excluding studies with small sample sizes, there are still high heterogeneity and publication bias among large sample studies. However, the results of subgroup analysis did not change after adjustment for publication bias by trim and fill method. Notably, through leave‐one‐out analysis, we found that 6 of the 7 literatures contributing most to heterogeneity were from large‐sample studies. These indicated that future large‐scale and well‐designed RCTs are still required to further examine our main findings.

In our study, we found that women were more likely to benefit from long‐term PE through subgroup analysis and meta‐regression. However, previous meta‐analysis (Szuhany et al., 2015) reported that the effect of long‐term regular exercise on peripheral BDNF levels was negatively correlated to the proportion of women included in the studies. Meanwhile, we also found that the elevation effect of PE on BDNF was only reflected in those older than 60 years. There is no doubt that gender and age are two important factors affecting the levels of BDNF. In addition, women and the elderly are also high‐risk groups of Alzheimer's disease and other diseases related to abnormal BDNF levels (Beam et al., 2018; Riedel et al., 2016). Weisbrod et al. (2019) also found that BDNF levels decreased in female rats after exposure to stress, but not in male mice. Our meta‐analysis could suggest that long‐term PE intervention might be effective for improving BDNF levels for people over 60‐years‐old and the female population. However, more females than males (*n* = 112/80) and more people over 60‐years‐old than under 60‐years‐old (*n* = 505/168) were included in the analyses, so future studies with equal sex and age ratios need to be replicated.

Based on current evidences, aerobic exercise has been proved to be successful in improving circulating BDNF (Cassilhas et al., 2012; Dinoff et al., 2016), while strength training seems to be mostly ineffective (Huang et al., 2014; Knaepen et al., 2010). A meta‐analysis also showed that aerobic exercise may contribute to increased levels of BDNF in neurological populations (Mackay et al., 2017). Consistent with previous studies, we also found that aerobic exercise, but not resistance training, increased circulating BDNF. Aerobic exercise is related to the improvement of endothelial function, insulin resistance, metabolic function, and cerebral blood flow, which are all associated with the increase of BDNF (Lemos et al., 2016; Zembron‐Lacny et al., 2016).

In our current study, we found that intensity of exercise did not influence the levels of BDNF. Most of studies admitted that the intensity of exercise is positively correlated with the increase of BDNF circulation levels. A systematic review reported that BDNF levels increase in an intensity‐dependent manner (Knaepen et al., 2010). Higher intensity of exercise is associated with hyperthermia, splenic response (Brunelli et al., 2012; Stewart et al., 2003), increased blood‐brain barrier permeability (Roh et al., 2017), and hypoxia. These are all related to the increase of BDNF release. Soya et al. found that acute treadmill running at low intensity (15 m/min) increased BDNF levels in the hippocampus of Wistar rats, but no increase was observed at moderate intensity (20 or 25 m/min) (Soya et al., 2007). Similarly, Gilder et al. (2014) found that serum BDNF increased by approximately 48% at 78% VO_2_max, but decreased at maximal exertion trial in healthy young men. Hence, the relationship between intensity of exercise and BDNF levels needs to be further investigated as a key point in clarifying the effect of PE on BDNF.

### 4.1 Limitations

There exist several limitations in this study. Firstly, 9 of the 21 included studies did not claim to adopt blinding, which resulted in the possibility of bias. The heterogeneity across studies and the limited sample size in some of the subgroups also made the interpretation of the results requiring to be cautious. In addition, some results showed publication bias, which might threaten the validity and interpretation of the effect; however, most original analyses remained unchanged after adjustment via trim and fill analysis. Lastly, only three included studies (Matura et al., 2017; Nilsson et al., 2020; Seifert et al., 2010) reported the results of fitness levels, making it impossible to explore the relationship between fitness levels and BDNF levels.

## 5 CONCLUSION

Taken together, both acute and long‐term PE significantly elevated circulating BDNF levels in healthy subjects. Long‐term aerobic exercise can lead to a more pronounced neurotrophic effect especially for female subjects or subjects over 60 years. Future large‐scale and high‐quality RCTs focusing on more detailed divisions of PE prescriptions and the importance of carefully considering the physiological response to PE will be of great necessity.

## CONFLICT OF INTEREST

The authors declare that they have no competing interests.

## AUTHOR CONTRIBUTIONS

All authors have contributed to the work in a meaningful way. Ya‐Hai Wang, Huan‐Huan Zhou, and Sidong Cui were involved in the conceptualization and design of the methodology. Ya‐Hai Wang and Huan‐Huan Zhou conducted the research, analyzed the data. Ya‐Hai Wang, Huan‐Huan Zhou, and Sidong Cui wrote the initial draft. Qiang Luo and Sidong Cui critically reviewed the manuscript. All authors agree with publication of the final version of the manuscript.

### PEER REVIEW

The peer review history for this article is available at https://publons.com/publon/10.1002/brb3.2544

## Supporting information

Supporting Information

## Data Availability

The data that support the findings of this study are available from the corresponding author upon reasonable request.
