# Supplementary material for: The effect of physical exercise on circulating brain‐derived neurotrophic factor in healthy subjects: A meta‐analysis of randomized controlled trials
Source: Brain Behav. 2022 Mar 11;12(4):e2544. doi: 10.1002/brb3.2544 (PMC9014996; doi:10.1002/brb3.2544)
Supplement: Supplementary file 1 — Supporting Information [file BRB3-12-e2544-s001.docx]

**Supplementary table 1**

Example search strategy for the PubMed database.

**((randomized controlled trial [Publication Type])OR (randomized[Title/Abstract]) OR (placebo[Title/Abstract])) AND ((Brain-Derived Neurotrophic Factor[Mesh]) OR (BDNF[Title/Abstract]) OR (Nerve Growth Factor[Mesh]) OR (NGF[Title/Abstract])) AND ((Exercise[Mesh]) OR (Exercises[Title/Abstract]) OR (Exercise, Physical[Title/Abstract]) OR (Exercises, Physical[Title/Abstract]) OR (Physical Exercise[Title/Abstract]) OR (Physical Exercises[Title/Abstract]) OR (Exercise, Isometric[Title/Abstract]) OR (Exercises, Isometric[Title/Abstract]) OR (Isometric Exercises[Title/Abstract]) OR (Isometric Exercise[Title/Abstract]) OR (Exercise, Aerobic[Title/Abstract]) OR (Aerobic Exercises[Title/Abstract]) OR (Exercises, Aerobic[Title/Abstract]) OR (Aerobic Exercise[Title/Abstract]) OR (Exercise Therapy[Mesh]) OR (Therapy, Exercise[Title/Abstract]) OR (Exercise Therapies[Title/Abstract]) OR (Therapies, Exercise[Title/Abstract]) OR (Exercise Movement Techniques[Mesh]) OR (Movement Techniques, Exercise[Title/Abstract]) OR (Exercise Movement Technics[Title/Abstract]) OR (Pilates-Based Exercises[Title/Abstract]) OR (Exercises, Pilates-Based[Title/Abstract]) OR (Pilates Based Exercises[Title/Abstract]) OR (Pilates Training[Title/Abstract]) OR (Training, Pilates[Title/Abstract]))**

Example search strategy for other databases.

**((****"randomized controlled trial" OR randomized OR placebo) AND (****"Brain-Derived Neurotrophic Factor" OR "BDNF" OR "Nerve Growth Factor" OR "NGF") AND (Exercise OR "Physical Exercise" OR "Exercise Therapy" OR "Exercise Movement Techniques" OR "Pilates-Based Exercises"))**

**Supplementary Table2 Grades of Recommendation, Assessment, Development and Evaluation (GRADE) quality of evidence**

| **Outcomes** | **Risk of**  **bias** | **Inconsistency** | **Indirectness** | **Imprecision** | **Publication**  **bias** | **Effect**  **size** | **Plausible**  **residual**  **confounding** | **Dose-response**  **gradient** | **GRADE**  **rating** |
| --- | --- | --- | --- | --- | --- | --- | --- | --- | --- |
| Acute PE on BDNF | 0 | -1^a^ | 0 | 0 | 0 | 0 | 0 | 0 | Moderate |
| Long-term PE on BDNF | 0 | -1^a^ | 0 | 0 | -1^b^ | 0 | 0 | 0 | Low |

^a.^ Significant and unexplained variability exists in the primary analysis; ^b^, p < 0.1 on Begg’s or Egger’s regression test.

Abbreviations: BDNF, brain-derived neurotrophic factor
